# Design, Response Rates, and Population Characteristics of a Cross-Sectional Study in Zanzibar, Tanzania

**DOI:** 10.2196/resprot.6621

**Published:** 2016-12-01

**Authors:** Maria Adam Nyangasa, Soerge Kelm, Mohammed Ali Sheikh, Antje Hebestreit

**Affiliations:** ^1^ Leibniz Institute for Prevention Research and Epidemiology-BIPS Bremen Germany; ^2^ Center for Biomolecular Interactions Bremen University of Bremen Bremen Germany; ^3^ Environmental Analytical Chemistry and Eco-toxicology Lab State University of Zanzibar Zanzibar United Republic of Tanzania

**Keywords:** cross-sectional study, anthropometric measures, blood pressure, biosamples, response rates, sub-Saharan Africa

## Abstract

**Background:**

Data on nutritional status and correlates of noncommunicable diseases are scarce for resource-poor settings in sub-Saharan countries. With the scope of a project, “Access to Food and Nutrition Status of the Zanzibari Population,” data for investigating public health questions were collected using proven measurement and laboratory standards.

**Objective:**

The present study aims at providing a descriptive overview of recruitment approaches, standardization, quality control measures, and data collection, with special attention to the design, responses, and participant characteristics of the overall project.

**Methods:**

A cross-sectional study across 80 randomly selected Shehias (wards) was conducted in 2013 in Unguja Island, Zanzibar. Examinations included all members living in 1 household, face-to-face interviews and anthropometric measurements (weight, height, mid-upper arm circumference, waist and hip circumference, and body composition) were assessed for all household members, blood pressure was taken from participants older than 2 years, and biosamples (urine and blood) from eligible household members were collected. Data collected from the core sample included sociodemographic data, nutritional status, and medical history (hypertension). Physical activity data was collected from a subsample of children between 3 and 16 years of age.

**Results:**

A total of 1314 participants (mean age 23.6 ± 18.9 years, 54.54% female) completed all anthropometric measurements and were included in the analysis. Out of which, 98.40% (1293/1314) completed the household member’s questionnaire, 93.32% (1229/1314) participants older than 2 years completed blood pressure measurements, and 64.31% (845/1314) blood samples were collected from participants older than 5 years. Underweight prevalence for the total study population was 36.53% (480/1314) with the highest prevalence in children under 14 years. Overweight and obesity was highest among females with the prevalence of 7.61% (100/1314) and 6.62% (87/1314), respectively; obesity was rare among male participants.

**Conclusions:**

The study provides valuable data to investigate the interplay of socioeconomic, demographic, environmental, physiological, and behavioral factors in the development of diet-related disorders in a representative sample of the Zanzibari population.

## Introduction

Food and nutrition insecurity is defined as the uncertain or limited access to safe, sufficient, and adequate food that is supported by an environment of adequate sanitation and health services to allow a healthy and active life [1]; it is a leading cause of morbidity and mortality worldwide. The United Nations Food and Agriculture Organization (FAO) estimates that approximately, 1 in 9 people was suffering from chronic undernourishment in 2012-2014, with a high prevalence in sub-Saharan African countries with low income [2]. Although some of these countries report to have adequate food at the national level, this does not guarantee food security at the household level [3]. Access to food in Zanzibar is one of the foremost food security problems for many Zanzibar households in both rural and urban areas. Access to food means individuals have adequate income or other sources to purchase or obtain levels of appropriate foods needed to maintain consumption of an adequate diet/nutrition level and are able to obtain these foods in socially acceptable ways [4].

Food insecurity has been linked to poor diet quality and has been found to have multiple negative health impacts beyond under nutrition, such as hypertension, obesity, and increased rates of gestational diabetes mellitus [5,6]. Also, data from mainland Tanzania show an increasing prevalence of overweight and obesity in urban, peri-urban, and rural areas [7-9]. In Zanzibar, education, food production, globalization, and sedentary lifestyle have noticeable effects on the health and nutrition status of the people. Like other developing countries, Zanzibar is undergoing a double burden of underweight and overweight/obesity [10] with a rapidly increasing number of noncommunicable diseases and associated risk factors. Data on nutrition and lifestyle factors and related determinants to assess the prevalence of cardiometabolic risk factors are scarce for resource-poor settings in sub-Saharan countries.

The project “Access to Food and Nutrition Status of the Zanzibari Population” comprises a population-based, cross-sectional survey in order to collect data for addressing these public health questions using proven measurement and laboratory standards [11,12]. The present study aims to describe the study design, field methods, and examination modules that were used to collect data in this representative study population. The present study will also present response proportions for all survey modules, prevalence estimates for underweight, overweight, and obesity for all study participants, and measures for data quality as well as giving a first glance on estimates of metabolic and nutritional markers of malnutrition for the study population. Further results on nutrition and health outcomes related to food access and food insecurity, diet, and biochemical indicators, as well as the potential determinants of nutritional status of the study participants are the subject of forthcoming publications.

## Methods

### Study Area

Zanzibar Island is located approximately 25 km off the coast of Mainland Tanzania. Zanzibar is comprised of 2 main Islands, Unguja and Pemba, with a projected population of 1.3 million people; almost 63% living in Unguja and 37% in Pemba [13]. Zanzibar Island has 2650 km^2^ of land area, of which two-thirds is coral-derived and one-third, where the population is concentrated, is good for agricultural production. Based on the food security situational analysis of 2006, Zanzibar produces 59% of its needs and the rest is from imports. The major foods produced are rice, cassava, bananas, sweet potatoes, maize, yams, and fish, while major imports includes rice, wheat flour, maize flour, and sugar [14].

Administratively, Zanzibar is divided into 5 regions, 3 in Unguja, and 2 in Pemba. Each region has 2 districts under the District Commissioner; each district is also subdivided into several smaller administrative units known as Shehias (equivalent to wards).

### Sampling

This baseline survey was carried out in 80 Shehias in Unguja. A two-staged sampling technique was used: (1) from a list of all 213 Shehias from the Tanzania population and housing Census 2010 [15], 80 Shehias were randomly selected using a Statistical Software procedure PROC SURVEYSELCT; and (2) participating households were randomly selected within each Shehia through the official Sheha’s (the Shehia’s administrative authority) registration where all members of each Shehia were registered according to household number and/or street name.

Households were defined as a group that shared meals and slept under the same roof [16], all members in a household constituted the target study population. The overall aim of the study was to estimate the prevalence of malnutrition in the Zanzibari population including possible correlates. As published prevalence estimates for malnutrition in the overall population are scarce, malnutrition prevalence among children under the age of 5 years (30% prevalence of chronic malnutrition in children <5 years) was taken from the Tanzanian Demographic and Health Survey 2010 [13]. Assuming a prevalence of approximately 30% malnutrition in children <5 years of age, a sample size of 323 children <5 years of age was needed to estimate 95% confidence interval with a precision of ±5%. The estimated sample size was determined based on the formula discussed by Lemeshow [17]. Given that each household takes care of 1 child <5 years of age and the reported average household size is 4.8 members [15], we estimated a final sample size of 1453 subjects when 4 households per Shehia were enrolled.

### Recruitment

Eligible households/participants were visited twice for recruitment and examination: during an evening visit, members of selected households were asked for agreement in participation, and on the subsequent morning they were examined. The field team randomly selected participating households by choosing any number from the household registration book. With the Sheha’s assistance, the selected households were visited for further information about the survey and enrollment, and to receive informed consent for all household members. Morning visits were for conducting all measurements, questionnaires, collection of morning spot urine and venous blood, distribution of accelerometers to eligible members, and providing feedback to all participants. Blood drawing and anthropometric measurements were carried out in fasting status.

All members of the selected households living together and eating from 1 pot were considered eligible. The main respondent was the head of household (father/mother). However, in the absence of the head of household, a guardian/caretaker of the house became the main respondent. In the case of polygamous families, all wives and their household members were also eligible for participation regardless of area of residence. One consent form was available per household; all participating members were given an anonymized identification number. Prior to the data collection process, all participants were verbally informed in Swahili on the study goals, purpose, target study group, contents, the amount of examinations involved, and that participation in the study was voluntary and consent could be withdrawn at any time without specifying any reason. Besides the verbal information given, all participants older than 16 years gave written consent and parents/guardians gave written consent on behalf of their children younger than 16 years. Participants signed 2 copies of the consent form, whereby 1 remained with them and the second was kept in the investigator's file on site. The study was approved by the Ethics Committees of the University of Bremen and of the Zanzibar Ministry of Health and the Zanzibar Medical Research and Ethics Committee in accordance with the ethical standards laid down in the Declaration of Helsinki and its later amendments. The consent forms were approved by the Institutional Ethics Committee.

As an incentive, every participating household received a Swahili feedback sheet with information on anthropometric measurements, body composition, blood pressure, and urine dip stick test results (for feedback purposes only).

### Anthropometric Measurements and Physical Examinations

The survey covered standard anthropometric measurements taken by trained fieldworkers following standardized procedures for anthropometric measurements, physical examination [11,18], and biosample collection and procession [12].

The measurement of body composition and body weight was carried out using an electronic scale (TANITA BC-420 SMA, Germany) to the nearest 0.1 kg. The surveyed area was a strict Muslim community; participants were covered with light clothing (“Kanga”). Like many similar studies [19,20], the weighing scale was tared with 1-kg clothing to give a zero reading before weighing the participant. At each instance, the actual weight of the participant was measured in fasting status and barefooted.

Height of participants who could stand alone was measured in accordance with international standards for anthropometric assessment and weight (kg) [11]. Recumbent length of young children under 2 years was measured using a measuring board (Seca 417 measuring board, UK); all measurements taken were recorded to the nearest 0.1 cm. Body mass index (BMI) was calculated by dividing weight in kilograms by height squared in meters (kg/m^2^) and then transformed to age- and sex-specific z-score. According to World Health Organization (WHO) [21] recommendations, BMI (adults) and BMI z-score (children) were used to define individuals who are underweight, normal weight, overweight, and obese.

Mid-upper arm circumference (MUAC) was measured at the mid-point of the left arm. Waist circumference (WC) was measured at the midway point between the lower rib margin and the iliac crest, and hip circumference (HC) was measured at the widest portion of the buttocks. Waist-to-hip ratio was obtained by dividing WC by HC. All circumferences were measured in standing position and while wearing light clothing using an inelastic measuring tape (SECA 201) to the nearest 0.1 cm.

A digital automatic blood pressure (BP) monitor (Omron T3) was used by trained fieldworkers to measure the systolic and diastolic BP according to a standardized procedure. Cuff length for BP measurement was determined according to the arm circumference, which was measured to the nearest 0.1 cm using an inelastic measuring tape. Participants were asked to rest for at least 15 minutes before measurements. Two measurements were taken at an interval of 5 minutes apart, plus a third measurement in case of a >5% difference in BP between the previous 2 readings. Use of antihypertensive medication and name(s) of medication were also recorded. For statistical analysis the lowest reading was considered. Hypertension was defined as a sustained high BP (systolic BP≥140 mm Hg or diastolic BP≥90 mm Hg) [22], or reported regular use of antihypertensive medication.

### Questionnaires

All forms and questionnaires were administered and well explained by trained fieldworkers in Swahili. Three main questionnaires (ie, head of household questionnaire, household members’ questionnaires, and young children questionnaire) were developed to collect data needed for the assessment. The questionnaires used were partly adapted from the Tanzania Demographic Survey 2010 [13] and Food and Nutrition Technical Assistance [23]. A partly validated head of household questionnaire was developed and administered to the head of the household (man/woman/guardian). This was used in collecting data on social, demographic, and economic indicators of the household: the ownership of livestock and other assets, sanitation, as well as information on household dietary behavior including expenditure and consumption. The household questionnaire also included a Swahili-translated version of Food Insecurity Experience questions [3], adapted from the original FAO 2007 [24]. Another tool used to measure food security was the Food Consumption Score, which was a composite score based on dietary diversity, food frequency, and relative nutritional importance of different food groups [25]. To measure nutritional quality and micronutrients adequacy of individual’s diets, an Individual Dietary Diversity Score was calculated based on the 14 food groups’ classification recommended by FAO (2007) [24]. Data derived with these instruments will be investigated in depth later and go beyond the scope of this study.

A (parental) proxy questionnaire was used to assess dietary diversity and consumption frequency of young children (0-24 months) addressing in particular breast-feeding habits, introduction to complimentary food, and a 24-hour dietary recall with food frequency questions adopted from the WHO [26].

### Biosamples

The study collected venous blood and morning urine samples from eligible participants in an overnight fasting status, all participants were informed during the evening visits to avoid eating or drinking anything (apart from water) prior to sample collection. According to several identified studies and guidelines [27], pediatric blood sample volume limits ranging from 1% to 5% of total blood volume within 24 hours is within the limits of minimal risks. In this study, venous blood was drawn from participants older than 5 years and the collection was restricted to 1% of the estimated total blood volume corresponding to approximately 8 mL in a child weighing 10 kg. Children between 5 and 10 years received an eutectic mixture of local anesthetics patch prior to the blood drawing. For healthy, nonpregnant adults weighing at least 50 kg, a maximum of 20.5-mL venous blood was drawn, this corresponds with other research guidelines for human blood drawing. Instructions and urine collection cups labelled with the identification number of the participants were given to each member of the household for morning urine collection. Morning urine of younger children was collected by the parents.

A 6-mL tube (4 mL for children) of ethylenediamine tetraacetic acid (EDTA) blood was collected from each participant and kept at 4°C and fractioning was done the same day in the laboratory. First, 0.5-mL whole EDTA blood was transferred into a 2-mL cryotube for glycated hemoglobin (HbA1c) analysis. Second, the EDTA blood was further separated after centrifugation for 10 minutes at 2500 *g*: this includes partitioning of the sample in 2 aliquots of plasma (0.5-1 mL) and 2 aliquots of red blood cells (RBC), as well as the isolation of 0.5-mL buffy coat white blood cells (WBC). Plasma, WBC, and RBC aliquots were sorted into separate cryoboxes and immediately stored at −80°C. From each participant, a 25-mL sample of urine was collected. Of the collected urine, 4 aliquots (5 mL) were transferred to a 5-mL vial. The urine samples were sorted into a separate cryobox for later analysis and stored in an upright position at −80°C. Blood and urine samples were analyzed for indicators of metabolic disorders and other health- and diet-related outcomes like blood glucose, HbA1c, cholesterol, triglycerides, low-density lipoprotein (LDL), insulin, leptin, C-reactive protein, urine albumin, and cytokines like tumour necrosis factor-α, inducible protein-10, interleukin (IL)-6, IL-8, IL-15, and IL-1 receptor antagonist.

### Physical Activity

To measure objective physical activity data, a subsample of 102 children and adolescents, aged between 3 and 16 years, were randomly selected from the participated households in 20 Shehias covering the rural, urban, and peri-urban areas. Participants wore a uniaxial accelerometer on the right side of the hip for 3-7 consecutive days. Raw data collected by the accelerometer were integrated into 15-second epochs using ActiLife software. Wear time and nonwear time were determined using the algorithms developed by Choi et al [28]. Nonwear time was defined as 90-consecutive minutes of 0 cpm, allowing up to a 2-minute interval of non-zero cpm. Information regarding the correct wearing of the accelerometer was explained to the parents and the participants prior to the initial wear. Both parents and participants were instructed to remove the accelerometer during sleeping, bathing, swimming, or at any point where they had full body contact with water. Parents checked and corrected the accelerometer on regular basis and used a diary to document the times and durations when the accelerometer was not worn or removed for specific reasons. These diaries were returned to the survey team mostly incomplete, and thus were not yet considered for analysis. Accelerometer measurements were included from children who wore the accelerometer for at least 3 days, including 1 weekend day, and for at least 6 hours per day. The duration of moderate-to-vigorous physical activity was determined according to the cut-offs of Evenson [29,30].

### Quality Control and Management

All measurements and methods followed standard operation procedures and were adopted from the Identification and prevention of the Dietary- and lifestyle-induced health EFfects in Children and InfantS study (IDEFICS) [11,12]. All instruments were developed in English and translated to Swahili and then back-translated to check for translation errors. The quality of translation and the relevance of the study and instruments were discussed with the team of experts from the Ministry of Agriculture under the department of Food and Nutrition and the United Nations Children's Fund in Unguja.

Field workers participated in local training in Zanzibar prior to the pretest; the fieldworkers were trained from basic interview techniques to the specific anthropometric measurements data collection as well as field methodology and sampling. Further training on laboratory procedures (ie, collection, processing, and storage of biosamples) and analysis were provided to laboratory technicians according to the IDEFICS study standards [12].

Prior to field work, a pretest was conducted in a convenience sample to test the wording flow, comprehension of the questions, and interviewing and anthropometric measurements techniques; the instruments were then modified accordingly. All instruments were finalized after the pretest of all measurements.

Fieldworkers were divided into 2 teams who used the same technical equipment and instruments for data collection to maximize comparability of data. Reliability measurement results were assessed by repeated measurements and swapping of the field team members on a regular basis. Quality control was applied daily during the field phase and included completeness, quality of documentation, and adherence to the study procedures. All numerical variables were entered twice, independently during data entry. In order to decrease measurement errors, subsamples of the study subjects were examined repeatedly to calculate the inter- and intraobserver reliability of anthropometric measurements. The protocol of the reliability measurements proposed that the manual set of anthropometric measurements (excluding those that were measured with an electronic and automated device) be taken in at least 20 children (10 girls and 10 boys, all primary school). Every observer measured each child 3 consecutive times within 1 hour in an extra sample. Analysis was performed according to Stromfai et al [11].

Quality control during biosample analysis comprised double analysis in most parameters where feasible (HbA1c, glucose, total cholesterol, triglycerides, LDL), and a third scan when the difference between the first 2 values was >10%. Other parameters were measured once, and reference samples (n=6) were scanned randomly in an independent laboratory in Germany controlling for interassay reproducibility of values. Interclass coefficient correlation between the 2 sets was calculated for the measurements of cholesterol, glucose NaF, HbA1c, HDL-cholesterol, LDL-cholesterol, LDL-HDL-quotient, and triglycerides.

## Results

### Household Characteristics

The household characteristic was comprised of the number of households that participated in the survey, household size, sex, marital status, highest head of household’s education level, source of income, as well as occupation (Table 1). A total of 239 households participated in the survey, more than half (131/239, 54.8%) were from rural areas with an average household size of 6 persons per household. Of the participated households, 62.8% (150/239) were headed by a male. Over half (124/226, 54.9%) were married in monogamy with the highest percentage from rural areas (69/124, 55.7%). The results further showed that more than three-quarters (184/224, 82.1%) of the household heads had attained some level of education, which means they could read and write; 17.9% (40/224) had no education. Of the respondents, 49.0% (117/239) had no regular income, and 45.6% (109/239) had at least 1 source of income to ensure stability of household food supplies, with a majority (73/109, 55.7%) of the respondents being from a rural area, and 5.4% (13/239) with more than 1 source of income. Primary sector (farmer, livestock, fishing, daily ages) was the main occupation group for the majority (73/215, 30.7%) of the household heads.

**Table 1:** General characteristics of head of households.

Characteristics		Rural	Urban	Peri-urban	Total
		n (%)	n (%)	n (%)	n (%)
Number of households		131 (54.8)	53 (22.2)	55 (23.0)	239
Average number of household members		6.0	8.1	7.1	6.7
**Gender of household head (n=239)**					
	Male	81 (61.8)	37 (69.8)	32 (58.2)	150 (62.8)
	Female	50 (38.2)	16 (30.2)	23 (41.8)	89 (37.2)
**Matrimonial status (n=226)**					
	Other^a^	20 (16.1)	13 (26.5)	10 (18.9)	43 (19.0)
	Monogamous	69 (55.7)	27 (55.1)	28 (52.8)	124 (54.9)
	Polygamous	35 (28.2)	9 (18.4)	15 (28.3)	59 (26.1)
**Education of the household head (n=224)**					
	None	28 (22.8)	5 (10.4)	7 (13.2)	40 (17.9)
	Primary	38 (30.9)	19 (39.6)	16 (30.2)	73 (32.6)
	Secondary	56 (45.5)	23 (48.0)	29 (54.7)	108 (48.2)
	Tertiary	1 (0.8)	1 (2.1)	1 (1.9)	3 (1.3)
**Source of income (n=239)**					
	None	50 (38.2)	35 (66.4)	32 (58.2)	117 (49.0)
	One	73 (55.7)	18 (34.0)	18 (32.8)	109 (45.6)
	More than one	8 (6.1)	0 (0)	5 (9.1)	13 (5.4)
**Occupation sector (n=215)**					
	None	40 (33.1)	27 (60.0)	26 (53.1)	93 (43.3)
	Primary^b^	52 (43.0)	5 (11.1)	9 (18.4)	66 (30.7)
	Secondary^c^	3 (2.5)	1 (2.2)	3 (6.1)	7 (3.3)
	Tertiary^d^	26 (21.5)	12 (26.7)	11 (22.5)	49 (22.8)

^a^Other includes single, divorced, widow, and cohabitation.

^b^Primary sector includes agriculture, fishing, mining, and forestry.

^c^Secondary sector includes craftsman and industry and construction work.

^d^Tertiary sector includes services and trade.

### Age and Gender Distribution of the Study Population

Table 2 represents age distribution of the study population by gender. The sample consisted of 1314 participants; age ranging from 0 to 95 years with a mean (SD) of 23.6 (SD 18.9) years. Participants were predominantly women (715/1314, 54.41%), of which 54.0% (386/715) were between 15 and 59 years. Working and reproductive age adults (15-59 years) comprised 50.15% (659/1314) of the total study participants, 15.60% (205/1314) of the population were children younger than 5 years, 28.01% (368/1314) were school-age children between 6 ad 14 years, and 6.62% (87/1314) were elderly participants.

**Table 2:** Age distribution of the study population stratified by gender.

Age group in years	Male	Female	Total
	n=599 (45.59%)	n=715 (54.41%)	N=1314 (100.00%)
0-2	27 (4.51)	36 (5.03)	63 (4.79)
3-5	67 (11.19)	75 (10.49)	142 (10.81)
6-14	192 (32.05)	171 (23.92)	363 (27.62)
15-59	273 (45.58)	386 (53.99)	659 (50.15)
60+	40 (6.68)	47 (6.57)	87 (6.62)

### Participation and Responses

**Figure 1:**
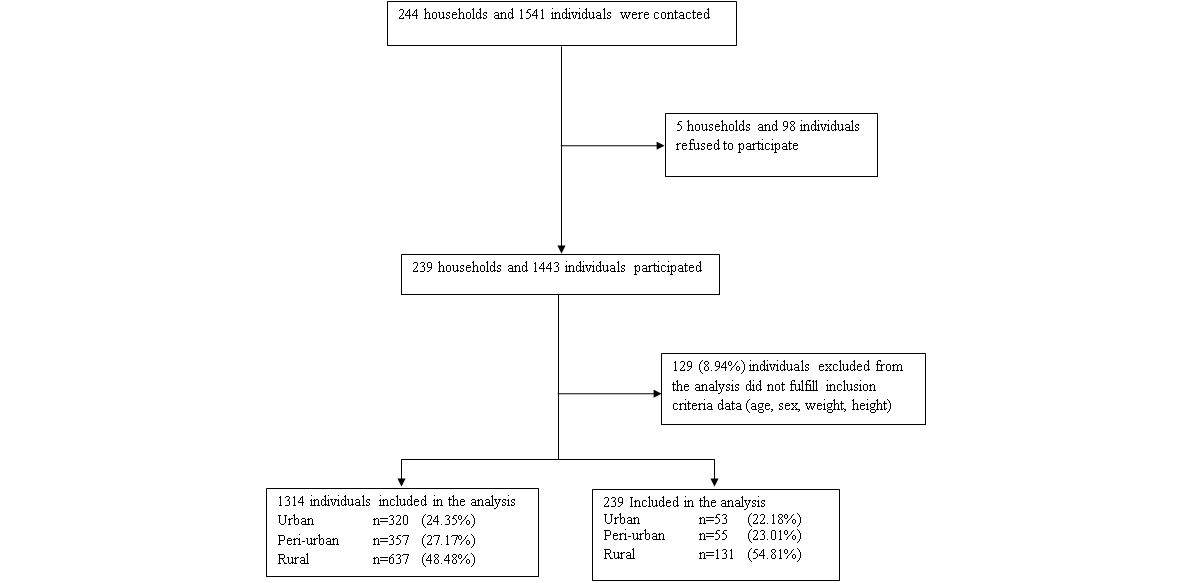
Flow diagram of participants from recruitment to analysis.

Out of 323 targeted households, 244 households were contacted, of which 97.9% (239/244) were included in the analysis and 2.1% (5/239) of households refused to participate. There were 1453 individuals targeted; however, due to high number of household members 1541 subjects were contacted, of which 1443 took part in 1 or more examination and questionnaire. Overall, 1314 participants fulfilled all inclusion criteria (weight, height/length, age, and gender) and were included in the analysis (Figure 1).

### Biomarkers in Urine and Blood

Table 3 presents proportions of analyzed biomarkers in urine and venous blood (≥5 years) stratified by age group. Approximately 63.5% (389/613) of the biosamples collected were from 15- to 59-year olds and the lowest proportion (8/585, 1.4%) was from children younger than 5 years, followed by elderly participants older than 60 years (58/613, 9.5%).

**Table 3:** Bio markers in urine and venous blood samples.

Biosample	Biomarker	<5 years	6-14 years	15-59 years	>60 years	Total
		n (%)	n (%)	n (%)	n (%)	n
**Urine**						
	Urine albumin-to-creatinine ratio^a^	85 (12.2)	202 (29.1)	353 (50.8)	55 (8.0)	695
**Blood**						
	Cholesterol^b^	6 (1.1)	152 (26.0)	372 (63.6)	55 (9.4)	585
	Glucose^b^	6 (1.0)	15.2 (26.0)	373 (63.7)	55 (9.4)	586
	Low-density lipoprotein^b^	7 (1.2)	15.2 (26.0)	372 (63.5)	55 (9.4)	586
	Triglycerides^b^	7 (1.2)	152 (26.0)	373 (63.5)	55 (9.4)	587
	C-reactive protein^c^	7 (1.2)	152 (26.0)	373 (63.5)	55 (9.4)	587
	Insulin^c^	8 (1.3)	158 (25.8)	389 (63.5)	58 (9.5)	613
	Leptin^c^	8 (1.3)	159 (25.9)	389 (63.5)	57 (9.3)	613
	IL^d^-1Ra^c^	8 (1.3)	158 (25.8)	389 (63.5)	58 (9.5)	613
	IL-6^c^	8 (1.3)	158 (25.8)	389 (63.5)	58 (9.5)	613
	IL-8^c^	8 (1.3)	159 (25.9)	389 (63.5)	57 (9.3)	613
	IL-15^c^	8 (1.3)	158 (25.8)	389 (63.5)	58 (9.5)	613
	Inducible protein-10^c^	8 (1.3)	158 (25.8)	389 (63.5)	58 (9.5)	613	
	Tumor necrosis factor-α^c^	8 (1.3)	158 (25.8)	389 (63.5)	58 (9.5)	613
	HbA1c^e,f^	7 (0.9	219 (26.9)	524 (64.3)	65 (8.0)	815
	Homeostatic model assessment-insulin resistance^g^	6 (1.0)	151 (26.1)	369 (63.6)	54 (9.3)	580

^a^Data available for some of the urine samples collected (at date of publication of manuscript).

^b^Measured in NaF plasma.

^c^Measured in blood serum.

^d^IL: interleukin.

^e^HbA1c: glycated haemoglobin.

^f^Measured in ethylenediamine tetraacetic acid blood (n=870).

^g^Homeostatic model assessment index = insulin (fasting, µU/mL) × blood glucose (fasting, mmol/L)/22.5.

### Individual Response for All Survey Modules

Core survey modules listed in Table 4 included questionnaires, anthropometric measurements, venous blood, BP, and a subsample for accelerometer use with children between 3 and 16 years. Besides the anthropometric measurement that had the minimum requirement inclusion for analysis (age, sex, height, weight), 100% response was not reached for other modules. The overall response rate for completing questionnaires was 98.40% (1293/1314); 64.31% (845/1314) for venous blood (restricted to participants ≥5 years), and 93.53% (1229/1314) for BP (participants ≥2 years). The use of an accelerometer was planned in a small subsample group of children between 3 and 16 years and was assessed in 95 children.

**Table 4:** Response of participants for all survey modules by age group.

Measurements/ examinations	Age group in years					Total	Overall response rate
	0-2	3-5	6-14	15-59	≥60		
	n (%)	n (%)	n (%)	n (%)	n (%)	n	%
Anthropometric measurements	63 (4.79)	142 (10.78)	364 (27.70)	659 (50.15)	86 (6.54)	1314	100
Household members questionnaire	60 (4.64)	142 (10.98)	358 (27.69)	648 (50.12)	85 (6.57)	1293	98.40
Venous blood^a^	-	8 (0.95)	233 (27.57)	537 (63.55)	67 (7.93)	845	64.31
Blood pressure^b^	-	124 (10.09)	364 (29.62)	655 (53.30)	86 (7.00)	1229	93.53
Accelerometer^c^	-	10 (10.53)	79 (83.16)	6 (6.32)	-	95	7.23
							

^a^Not measured in children below 5 years.

^b^Not measured in young children below 2 years.

^c^Measured only in a subsample of children and adolescents between 3 and 16 years (n=95).

### Prevalence of Malnutrition

Table 5 represents the prevalence of nutritional status according to gender and age group of the entire study population. The overall prevalence of underweight, overweight, and obesity was 36.45% (479/1314), 13.09% (172/1314), and 8.14% (107/1314), respectively; 42.31% (556/1314) of participants had normal weight. A higher proportion 70.24% (144/205) of children below the age of 5 years were underweight, more boys were underweight compared with girls. Adult females ages 15-59 years were more obese (71/386, 18.39%) compared with males of the same age group. One-third (28/87, 32.18%) of the elderly participants, both male and female and 60+ years, were overweight, and 16.09% (14/87) were obese.

**Table 5:** Prevalence of nutritional status stratified by gender and age group.

Gender/age group		Underweight	Normal weight	Overweight	Obese	Total
		n (%)	n (%)	n (%)	n (%)	n
**Male**						
	0-5 years	69 (73.40)	22 (23.40)	2 (2.12)	1 (1.06)	94
	6-14 years	120 (62.18)	69 (35.75)	1 (0.52)	3 (1.55)	193
	15-59 years	56 (20.51)	149 (54.58)	56 (20.5)	12 (4.40)	273
	60+ years	7 (17.95)	14 (35.90)	13 (33.33)	5 (12.82)	39
	Total male	252 (42.07)	254 (42.40)	72 (12.02)	21 (3.51)	599
**Female**						
	0-5 years	75 (67.57)	27 (24.32)	4 (3.60)	5 (4.50)	111
	6-14 years	93 (54.39)	63 (36.84)	13 (7.60)	2 (1.17)	171
	15-59 years	53 (13.73)	194 (50.26)	68 (17.62)	71 (18.39)	386
	60+ years	6 (12.77)	17 (36.17)	15 (31.91)	9 (19.15)	47
	Total female	227 (31.75)	301 (42.10)	100 (13.16)	87 (12.17)	715
**Total**						
	0-5 years	144 (70.24)	49 (23.90)	6 (2.93)	6 (2.93)	205
	6-14 years	213 (58.68)	132 (36.36)	14 (3.86)	4 (1.10)	363
	15-59 years	109 (16.54)	343 (52.15)	124 (18.82)	83 (12.59)	659
	60+ years	13 (14.94)	32 (36.78)	28 (32.18)	14 (16.10)	87
	Overall	479 (36.45)	556 (42.31)	172 (13.10)	107 (8.14)	1314

### Quality Indicators

Results of inter- and intraobserver technical error of measurements (TEM) were generally low. Interobserver reliability as assessed by the R for repeated measurements of height, MUAC, WC, and HC was above 99% for all participants (Table 6). TEM% provides comparability of quality between measurements.

**Table 6:** Inter- and intraobserver technical error of height and circumferences of the arm, waist, and hip in an extra sample.

	Interobserver				Intraobserver (n=30)			
	Mean	TEM^a^	TEM%	R%	Mean	TEM	TEM%	R%
Height	1.36507	0.003524036	0.25816	99.98	1.27973	0.003570410	0.27900	99.99
MUAC^b^	0.21628	0.001305582	0.60365	99.95	0.20203	0.004684696	2.31877	99.35
WC^c^	0.66582	0.001857418	0.27897	99.98	0.59423	0.007834197	1.31837	99.73
HC^d^	0.76888	0.007254823	0.94356	99.86	0.70237	0.004697209	0.66877	99.94

^a^TEM: technical error of measurements.

^b^MUAC: mid upper arm circumference.

^c^WC: waist circumference.

^d^HC: hip circumference

## Discussion

### Participation and Response

Currently, there are no existing studies that collected anthropometric, biological, socioeconomic, and nutritional data in a representative study sample in randomly selected households in Zanzibar following standardized procedures. This study was successful in setting up a cross-sectional survey of over 1400 participants in 80 Shehias across Unguja. The study included young children to elderly participants ages 0-95 years. The response rate was higher than expected. This is most probably due to multiple reasons: (1) a good training of the field workers in giving out information to participants, (2) feedback on results that are normally not affordable if requested by a physician, and (3) support through the Shehas who clearly had an interest in the results of the study. Prior to the start of the survey, all Shehas were invited to a presentation of the aims and content of the study (examinations) and to request official approval and support by the Shehas. Information on this meeting was disseminated in the local media, describing the aims of the study, which led to an overall high acceptance among participating families. Further, all the measurements and examinations were conducted at the participant’s homes and at their convenient time, having the advantage that all eligible individuals could be enrolled regardless of child care or (home) work. Our finding of an average household size of 6.7 compared with the officially reported average household size of 4.8 [15] supports that an official dissemination of study aims and measurements is a useful strategy to enhance response proportions for complete households. Also, the feedback sheets on personal measurements were appreciated and were useful to enhance response rates at family and community level. A feedback on all parameters of interest was promised to all Shehas, researchers, politicians, and stakeholders of health services prior to the survey and was presented 1 year after survey termination at a 2-day workshop in Zanzibar. Also, this workshop and the results were disseminated through the local media (television, newspaper) in order to give feedback to the general population.

However, approximately 11.29% (129/1443) of participants were excluded from the study for not fulfilling all criteria (weight, height/length, age, and sex) needed for analysis. This was due to respondents not knowing their exact date of birth and/or not having any records. Comparable to our results, Korkalo et al [31] encountered a similar problem in their study. Reasons for refusing anthropometric measurements were: pregnancy, illness/weakness, or fear in young children <2 years; a similar challenge was discussed in a previous study [32].

### Quality Control

Anthropometric measurement error is unavoidable and should be minimized by high-quality standards regarding every aspect of the data collection process. In all cases intra- and interobserver reliability for all measurements was greater than 99%, these results are very similar and even better than other reported studies [11,33,34]. Comparison of results from this study to previous investigated TEM and R values indicated that height and MUAC were similar and within the range with the reference values recommended for intraobserver values by Ulijaszek and Kerr [33]. Interobserver reliability for all circumferences was above 99%, other researchers reported ranges for MUAC 94%-100% , WC 86%-99%, and HC 68%-99% [33]. Compared with above results, the present data prove a high degree of accuracy during examination. We conclude that the local training and using the rigorous standardization approach facilitated the collection of accurate and comparable data.

For quality control of the biosamples, data collection, processing, and storage, protocol was adapted from IDEFICS study [32] that applied a quality management system for the collection of biological sample in epidemiology study; the system was developed and introduced by Peplies et al [12]. The results of the validation study and the very high level of agreement we observed underlines the need for standardizing biosample collection, procession, storage, and analysis, as well as for controlling the reliability of values obtained in order to repeat analysis if needed.

### Anthropometry and Nutritional Status

This study provides information on the anthropometry and nutritional status of all members of household included in the survey. The comparison across Shehia, age groups, and gender facilitate focused nutrition interventions in these aspects. Although the mean BMI for both male and female was within the normal range (18.5-24.9 kg/m^2^), both problems of underweight and overweight/obese were prevalent for both male and female. Among all study participants, 36.37% (479/1314) were considered to be underweight with a slightly higher prevalence in males (252/1314, 19.13%) compared with their female counterpart (227/1314, 17.28%). Of the 479 underweight participants, the highest proportion (213/479, 44.5%) was among school age children between 6 and 14 years. This result is almost similar to a study conducted in Nigeria (43.5%) for children between 9 and 12 years [35], and higher than studies conducted in India (38.4%) for children between 5 and 15 years [36], Mauritius (37.0%) for children between 8 and 12 years [37], and Uganda (13.0%) for children between 9 and 15 years [38]. The prevalence of underweight among young children <5 years was 70.1% (144/204), which is higher than reported in other studies conducted in sub-Saharan Africa for children <5 years in Tanzania Mainland (16%), Zanzibar (19%), Kenya (11.8%), and Cameroon (12.9%), respectively [13,39-41]. Although there are differences in sampling and target age groups, but nevertheless, the prevalence of underweight in our population-based survey among children <5 years and school age children 6-14 years is clearly higher than the above mentioned studies.

Overall prevalence of overweight and obesity is 13.1% and 8.1%, respectively, with the highest proportion in adult female participants ≥15-years old compared with their male counterparts. Prevalence of overweight and obesity for females 15- to 59-years old is 17.6% and 18.4%, respectively, and for elderly people above 60 years is 31.9% and 19.1%, respectively. This is found to be higher than that of the male participants, but lower than several studies conducted in Africa [42-45]. A higher prevalence of overweight and obesity among women has also been reported in other studies [13,44,45].

### Strengths and Limitations

Age and gender are the primary basis for demographic classification and also very important variables in the study of mortality, fertility, and marriage [13]. As reported in the comprehensive food security and vulnerability analysis [46], Tanzania overall has a very young population due to its fertility rate in the past. The present study shows a similar trend for Unguja Island with approximately 43% of the population being <15 years, of which approximately 15% are <5 years of age. Unfortunately, the response rate for children <5 years is less than the expected sample size; this is due to the higher household size and that the priority of enrollment was given to entire families rather than on age. Nevertheless, we are confident that the overall sample size of 1314 individuals will enable us to investigate the aimed research question with sufficient statistical power.

A strong cooperation and support by the Shehas of the study Shehias facilitated an easy and problem-free collection of data. Standardized approaches and clear documentation of the field information made it easy and required less time for data editing and entry. This was possible due to a successful training of the survey team prior the field trips. Feedback results of the anthropometric measurements, urine test results, and BP were well appreciated incentives, which not only motivated the participants to take part, but also provided them with information on their health status immediately after the measurements and free of charge. One of the methodological challenges was taking anthropometric measurements from women and young children. Women wore several layers of light clothing during measurements, might have affected the measurements taken, and children were mostly playful and unstable during measurements. However, these challenges are unlikely to change the findings and prevalence because 1 kg for clothing was deducted from weight measurements for all participants. Another challenge encountered was that most of the participants did not know or could not remember their precise date, month, and/or year of birth; however, this problem was solved with the help of an event calendar developed by the field team prior to the field work. Despite these limitations in the method and data collection, recruitment and participation was very successful and data collected was a good representative of the population studied.

### Conclusions

We can conclude that a rigorous standardization process and comprehensive training facilitated the collection of accurate and comparable anthropometric and biodata. The study provides valuable data to investigate the interplay of socioeconomic, demographic, environmental, physiological, and behavioral factors in the development of diet-related disorders in a representative sample of the Zanzibari population.

Further, investing time and endeavouring into a dissemination strategy involving important gatekeepers is a useful strategy to enhance response rates and acceptance in the study population. Transparency of population-based survey results at the individual, community, and governmental level are recommended at any time.

## Abbreviations

BMI: body mass index
BP: blood pressure
EDTA: ethylenediamine tetraacetic acid
FAO: Food and Agriculture Organization
HbA1c: glycated hemoglobin
HC: hip circumference
IDEFICS: Identification and prevention of the Dietary- and lifestyle-induced health EFfects in Children and InfantS study
IL: interleukin
LDL: low-density lipoprotein
MUAC: mid upper arm circumference
RBC: red blood cells
TEM: technical error of measurements
WBC: white blood cells
WC: waist circumference
WHO: World Health Organization
